# Stool cultures at the ICU: get rid of it!

**DOI:** 10.1186/s13613-018-0358-x

**Published:** 2018-01-18

**Authors:** Carolin F. Manthey, Darja Dranova, Martin Christner, Laura Berneking, Stefan Kluge, Ansgar W. Lohse, Valentin Fuhrmann

**Affiliations:** 1 First Department of Internal Medicine and Gastroenterology, University Hospital Hamburg-Eppendorf, Martinistr. 52, 20246 Hamburg, Germany; 2 Department of Intensive Care Medicine, University Hospital Hamburg-Eppendorf, Hamburg, Germany; 3 Department of Microbiology, University Hospital Hamburg-Eppendorf, Hamburg, Germany

**Keywords:** Diarrhea, Stool culture, Critically ill, *Clostridium difficile*, *Campylobacter* spp., *Salmonella* spp.

## Abstract

**Background:**

Stool cultures for *Campylobacter*, *Salmonella* and *Shigella* and/or *Yersinia* spp. are frequently ordered in critically ill patients with diarrhea. The aim of this study is to analyze the diagnostic yield in a large cohort of critically ill patients. Therefore, we performed a cohort study at the Department of Intensive Care Medicine of a University Hospital (11 ICUs).

**Results:**

From all patients who were admitted to the ICU between 2010 and 2015, stool cultures were taken from 2.189/36.477 (6%) patients due to diarrhea. Results of all stool cultures tested for *Campylobacter*, *Salmonella* and *Shigella* and/or *Yersinia* spp. were analyzed. Overall, 5.747 tests were performed; only six were positive (0.1%). In four of these, *Campylobacter* spp. were detected; diarrhea started within 48 h after ICU admission. Two patients with *Salmonella* spp. detection were chronic shedders. On the contrary, testing for *Clostridium difficile* via GDH- and toxin A/B-EIA yielded positive results in 179/2209 (8.1%) tests and revealed 144/2.189 (6.6%) patients with clinically relevant *C. difficile* infection.

**Conclusions:**

Stool testing for enteric pathogens other than *C. difficile* should be avoided in ICU patients and is only reasonable when diarrhea commenced less than 48 h after hospital admission.

## Background

Diarrhea is a common problem in critically ill patients. Reported prevalence varies from 2 to 95% [1] mostly owing to heterogeneous case definitions. The most common risk factors associated with diarrhea in this patient population are side effects of medications, dysbiosis due to antibiotic therapy, enteral feedings and enteric infections [2] as well as severe disease accompanying multiple organ dysfunction syndrome (MODS) [3]. *Clostridium difficile* infections (CDI) affect a significant amount of hospitalized patients [4], especially critically ill patients are at risk, whereas numbers for other enteric infections in this patient group remain elusive.

Current guidelines are recommending stool cultures for enteropathogenic bacteria for hospitalized patients with fever (> 101 °F/38.3 °C) and diarrhea that developed within 72 h after hospital admission [5]. However, this is not valid for critically ill patients as they have a significantly higher risk of complications (e.g., elderly, immunocompromised, certain comorbidities) [6]. Therefore, it is common practice to, even repeatedly, test for enteropathogenic bacteria in critically ill patients developing diarrhea [7]. This practice is cost-consuming, and in the case of *C. difficile* infections, repeated testing does not even increase diagnostic yield [7]. The aim of this study is to assess the clinical impact of diagnostic stool testing in critically ill patients.

## Methods

We performed a retrospective analysis of all stool samples collected during January 2010 and September 2015 in 11 ICUs (specialized for heart surgery, cardiology, internal medicine, neurosurgery, neurology and surgery, in addition to five interdisciplinary wards) of the Department of Intensive Care at the University Medical Center Hamburg. In addition to *C. difficile* toxin testing, cultures for enteropathogenic bacteria had consistently been requested for ICU patients with diarrhea during that time. Stool cultures for *Campylobacter* (modified Karmali agar), *Salmonella*–*Shigella* (MacConkey agar, Salmonella–Shigella agar, xylose lysine deoxycholate agar and selenite enrichment broth) and enteropathogenic *Yersinia* (CIN agar) had been performed by the Department of Medical Microbiology according to current practice guidelines by the German Society for Hygiene and Microbiology [8], while the C. diff Quik Chek Complete EIA (TechLab; Blacksburg, VA, USA) had been used for *C. difficile* glutamate dehydrogenase antigen (GDH) and toxin A/B testing of non-formed stool samples as recommended by the manufacturer. Only liquid stools were analyzed as a definition of diarrhea by C. diff Quik Chek, whereas culturing for other enteropathogenic bacteria was performed also from solid specimens. The number of stool passages per day for each patient was not available.

Anonymized data were extracted from our hospital and laboratory information systems.

The study was performed in accordance with the local regulations of the ethics committee (General Medical Council Hamburg, Ärztekammer Hamburg, reference number WF 11/16). Data analysis was performed anonymously, and informed consent was waived by the ethics committee for this retrospective study.

## Results

**Fig. 1:**
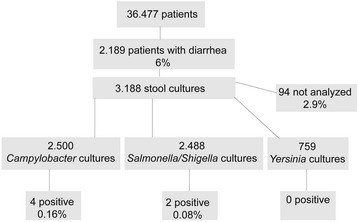
Flowchart of stool culture analysis at 11 ICUs 2010–2015 at the University Hospital Hamburg-Eppendorf

During the study period, 3188 stool samples from 2189 ICU patients were sent to the microbiology laboratory (Fig. 1). Patients tested represented 6.0% of all patients admitted (2010–2015: 41.415 admissions of 36.477 patients). Ninety-four samples (2.9%) were rejected by the laboratory due to several reasons (solid stool, scarcity of material).

Overall, 5.747 tests were performed; only six were positive (0.1%).

Only four out of 2500 *Campylobacter* cultures yielded a positive result (0.16% of all tests performed, 0.01% of all patients admitted); three samples were positive for *Campylobacter jejuni*, and one for *Campylobacter coli*. Symptoms in all four patients with *Campylobacter* infection had started less than 48 h after hospital admission implying that these patients had community acquired diarrhea. None of these patients received specific antibiotic therapy as symptoms had already resolved spontaneously. Length of ICU stay after diagnosis of *Campylobacter* enteritis was not increased, since all four patients were transferred to the normal ward the next day. None of the patients with *Campylobacter* enteritis died during their hospital stay.

Testing for *Salmonella* and *Shigella* (Salmonella–Shigella culture) was done for 2488 samples (1678 patients), two of these were positive for *Salmonella enterica* (0.08% of all tests performed, 0.005% of all patients), and none were positive for *Shigella*. *Salmonella enterica* was detected in the accidental submission of stool from two chronic shedders 20 and 23 days after hospital admission. The patients were not treated antimicrobially due to lack of symptoms. Detection of infection did not delay hospital dismissal or further transfer to another hospital. The main diagnoses in patients with *Campylobacter* enteritis were pneumonia (patient no. 1), postoperative due to thymectomy (patient no. 2) and vascular graft (patient no. 3), as well as epileptical attack (patient no. 4). Underlying comorbidities in these patients included Korsakov syndrome (patient no. 1), myasthenia gravis, arterial hypertension (patient no. 2), chronic obstructive pulmonary disease, reflux disease, sigma diverticulitis (patient no. 3), and cerebral infarction and arterial hypertension (patient no. 4). Patients with *Salmonella* detection were not on any immunosuppressive medication; however, one patient was suffering from head and neck cancer requiring multiple surgeries. The other patient with *Salmonella* infection was admitted due to subarachnoid hemorrhage.

None of the 759 samples tested for enteropathogenic *Yersinia* spp. yielded positive results (Fig. 1).

Regarding ICU characteristics, all six patients tested positive for *Salmonella* or *Campylobacter* required vasopressor support (mean 5.7 days ± 6.1) during their stay. Only one patient received vasopressor infusion on the day of the diagnosis, and the charts do not indicate that the dosage of vasopressor infusion was increased in patients due to diarrhea. Five out of six patients were in need of mechanical ventilation, although only one patient was on mechanical ventilation at the time of diagnosis. Length of mechanical ventilation was < 3 days in all three patients with *Campylobacter* enteritis. None of the patients required renal replacement therapy.

In contrast, *C. difficile* toxin testing yielded positive results in 242 (GDH antigen only) and 179 (GDH antigen and toxin A/B) of 2209 samples from 1654 patients (11.0 and 8.1% of all tests, 0.7 and 0.5% of all patients), the latter group fulfilling criteria for clinically relevant *C. difficile* infection in symptomatic patients [9]. 52/144 (36.1%) episodes of CDI occurred within 48 h of ICU admission, and 108/144 (75%) were antibiotic-associated.

**Fig. 2:**
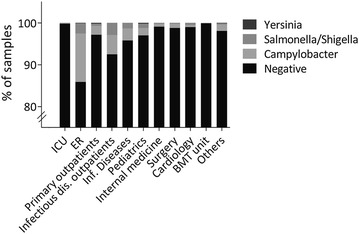
Results of all stool cultures taken during 2010–2015 at the University Center in Hamburg-Eppendorf according to patients’ site where the stool sample was taken; depicted as % of all analyzed specimens; specimens taken per week: 1–5 (surgery), 5–10 (infectious diseases, pediatrics, BMT), 10–15 (ICU, ER, outpatients, cardiology, others), 25–30 (internal medicine). *ICU* intensive care unit, *ER* emergency room, *BMT unit* bone marrow transplant unit

Regarding stool tests in the overall hospital community, results between 2010 and 2015 in all departments of our hospital are illustrated in Fig. 2. Patients presenting to the emergency room (ER) with diarrhea showed the highest rates of infection with *Campylobacter* spp. (11.6% of all analyzed specimens) and *Salmonella* or *Shigella* spp. (2.4%). Also, patients presenting to the infectious diseases department showed higher infection rates with *Campylobacter* (4.6%) and *Salmonella* or *Shigella* spp. (2.9%). Infection rates with *Yersinia* were overall very low with a maximum rate of infection in general outpatients (0.2% of samples). The lowest rate of bacterial infection in patients presenting with diarrhea was observed in patients treated at the ICU and bone marrow transplant unit.

Economic impact was estimated based on national medical fee schedules in Germany. Considering usual local discounts, we conferred costs of 20 € for enteric bacteria stool culture and 10 € for *C. difficile* testing adding up to 50,000 € (enteric bacteria stool culture) and 22,090 € (*C. difficile*), total 72,090 €.

## Discussion

Our data from 5 years of extensive testing for enteric bacterial pathogens in adult critically ill patients show that *C. difficile* can be regarded as the only relevant bacterial cause of gastrointestinal infection in critically ill patients with diarrhea. The very low yield of stool cultures from patients at the ICU observed in our study confirms current guideline recommendations for non-critically ill patients [5, 10] to limit testing in cases of suspected nosocomial gastrointestinal infection to *C. difficile.* Infections with *C. difficile* pose an important risk of increased mortality in patients at the ICU, and diagnostics regarding infection have to be conducted in case of suspected infection [8]. We can show that the highest rates of infections with enteric pathogens other than *C. difficile* are observed in patients presenting to the emergency room. *Campylobacter* spp. are the most important bacterial pathogen detected in outpatients; this reflects national reports by the Robert Koch Institute.

Simple stratification by time since hospital admission would have reduced the number of stool cultures at the ICU by more than 75% (72 h) or 80% (48 h) while still allowing for the detection of all four *Campylobacter* patients, which presented with diarrhea within a 48-h time frame. Application of more sophisticated rejection rules (considering patient age, comorbidities or non-diarrheal manifestations) would have decreased test numbers without significant effects on yield. Former reports have also demonstrated that stool cultures taken after the third day of hospitalization in non-critically ill adult patients only yielded positive results in 0.2% [11]–0.6% [12].

We present data applicable to management of diarrhea in critically ill patients at the ICU and hereby confirm previous findings [13]. Our findings undermine the fact that critically ill patients with diarrhea should be managed in exactly the same way as patients on the normal ward to reduce the number of unnecessary stool cultures. The comparably high costs of stool cultures do not seem justified given the low yield and limited consequences. Due to the retrospective study design of our single-center study, further studies are needed to confirm our findings.

## Conclusion

Our data indicate that diarrhea in patients at the ICU is seldom caused by enteropathogenic bacteria. Critically ill patients should be assessed for alternative reasons underlying diarrhea and be tested once to exclude infection with *C. difficile* and in cases of suspected outbreaks for viruses but not for other enteropathogenic bacteria as long as symptoms commenced > 48 h after admission.

## References

[1] Thibault R, Graf S, Clerc A, Delieuvin N, Heidegger CP, Pichard C (2013). Diarrhoea in the ICU: respective contribution of feeding and antibiotics. Crit Care.

[2] Wiesen P, Van Gossum A, Preiser JC (2006). Diarrhoea in the critically ill. Curr Opin Crit Care.

[3] Wei Y, Yang J, Wang J (2016). Successful treatment with fecal microbiota transplantation in patients with multiple organ dysfunction syndrome and diarrhea following severe sepsis. Crit Care.

[4] Manthey CF, Eckmann L, Fuhrmann V (2017). Therapy for *Clostridium difficile* infection—any news beyond Metronidazole and Vancomycin?. Exp Rev Clin Pharmacol.

[5] Riddle MS, DuPont HL, Connor BA (2016). ACG clinical guideline: diagnosis, treatment, and prevention of acute diarrheal infections in adults. Am J Gastroenterol.

[6] Bauer TM, Lalvani A, Fehrenbach J (2001). Derivation and validation of guidelines for stool cultures for enteropathogenic bacteria other than *Clostridium difficile* in hospitalized adults. JAMA.

[7] Deshpande A, Pasupuleti V, Patel P (2011). Repeat stool testing to diagnose *Clostridium difficile* infection using enzyme immunoassay does not increase diagnostic yield. Clin Gastroenterol Hepatol.

[8] Mauch HPA, Herrmann M, Kniehl E, Kist M (2013). MIQ 09: Gastrointestinale Infektionen Qualitätsstandards in der mikrobiologisch-infektiologischen Diagnostik.

[9] Planche TD, Davies KA, Coen PG (2013). Differences in outcome according to *Clostridium difficile* testing method: a prospective multicentre diagnostic validation study of *C. difficile* infection. Lancet Infect Dis.

[10] Hagel S, Epple HJ, Feurle GE (2015). S2k-guideline gastrointestinal infectious diseases and Whipple’s disease. Z Gastroenterol.

[11] Le Guern R, Loiez C, Grandbastien B, Courcol R, Wallet F (2013). Performance of stool cultures before and after a 3-day hospitalization: fewer cultures, better for patients and for money. Diagn Microbiol Infect Dis.

[12] Valenstein P, Pfaller M, Yungbluth M (1996). The use and abuse of routine stool microbiology: a College of American Pathologists Q-probes study of 601 institutions. Arch Pathol Lab Med.

[13] Tirlapur N, Puthucheary ZA, Cooper JA (2016). Diarrhoea in the critically ill is common, associated with poor outcome, and rarely due to *Clostridium difficile*. Sci Rep.

